# Streamlining DNA Barcoding Protocols: Automated DNA Extraction and a New *cox1* Primer in Arachnid Systematics

**DOI:** 10.1371/journal.pone.0113030

**Published:** 2014-11-21

**Authors:** Nina Vidergar, Nataša Toplak, Matjaž Kuntner

**Affiliations:** 1 Institute of Biology, Scientific Research Centre of the Slovenian Academy of Sciences and Arts, Ljubljana, Slovenia; 2 Centre for Behavioural Ecology & Evolution, College of Life Sciences, Hubei University, Wuhan, China; 3 National Museum of Natural History, Smithsonian Institution, Washington, DC, United States of America; 4 Omega d.o.o., Ljubljana, Slovenia; 5 Molecular Virology lab, International Centre for Genetic Engineering and Biotechnology–ICGEB, Trieste, Italy; The New York Botanical Garden, United States of America

## Abstract

**Background:**

DNA barcoding is a popular tool in taxonomic and phylogenetic studies, but for most animal lineages protocols for obtaining the barcoding sequences—mitochondrial cytochrome C oxidase subunit I (*cox1* AKA CO1)—are not standardized. Our aim was to explore an optimal strategy for arachnids, focusing on the species-richest lineage, spiders by (1) improving an automated DNA extraction protocol, (2) testing the performance of commonly used primer combinations, and (3) developing a new *cox1* primer suitable for more efficient alignment and phylogenetic analyses.

**Methodology:**

We used exemplars of 15 species from all major spider clades, processed a range of spider tissues of varying size and quality, optimized genomic DNA extraction using the MagMAX Express magnetic particle processor—an automated high throughput DNA extraction system—and tested *cox1* amplification protocols emphasizing the standard barcoding region using ten routinely employed primer pairs.

**Results:**

The best results were obtained with the commonly used Folmer primers (LCO1490/HCO2198) that capture the standard barcode region, and with the C1-J-2183/C1-N-2776 primer pair that amplifies its extension. However, C1-J-2183 is designed too close to HCO2198 for well-interpreted, continuous sequence data, and in practice the resulting sequences from the two primer pairs rarely overlap. We therefore designed a new forward primer C1-J-2123 60 base pairs upstream of the C1-J-2183 binding site. The success rate of this new primer (93%) matched that of C1-J-2183.

**Conclusions:**

The use of C1-J-2123 allows full, indel-free overlap of sequences obtained with the standard Folmer primers and with C1-J-2123 primer pair. Our preliminary tests suggest that in addition to spiders, C1-J-2123 will also perform in other arachnids and several other invertebrates. We provide optimal PCR protocols for these primer sets, and recommend using them for systematic efforts beyond DNA barcoding.

## Background and Objectives

**Figure 1:**
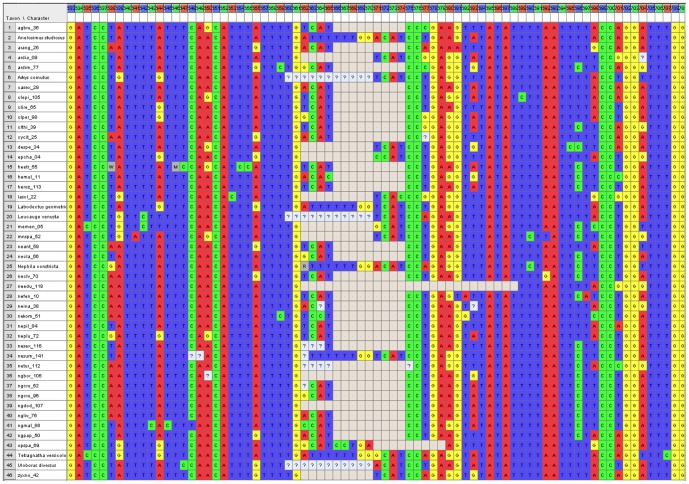
An artificial indel region in the alignment of *cox1* sequences between the Folmer region and the C1-J-2183/C1-N-2776 extension. Such indel region arising due to incomplete or poor reads, commonly hampers the accuracy of phylogenetic analyses.

DNA barcoding in animals routinely uses the mitochondrial gene cytochrome *C* oxidase subunit I (*cox1*, also CO1) [1]–[8] and the same gene is also among the usual markers employed in phylogenetic, genetic and genomic analyses [9]–[27]. In spiders and other arachnids, the standard barcoding region — 650 base pair long fragment of *cox1 —* is usually targeted with the use of a few selected primer pairs (Table 1). For phylogenetic and phylogenomic analyses, however, a longer stretch of *cox1* is targeted [9], [13]–[14], [28]–[29], but the primer pairs, or combinations of them yielding these nucleotide data may provide only limited amplification success [12], [30]–[32] whose outcome are data deficient alignments between two targeted *cox1* regions such as between those targeted by the Folmer region [33] and the C1-J-2183/C1-N-2776 extension [28], [30] (Fig. 1). Such indel region, arising through incomplete or poor reads, is artificial due to simple lack of data, and may reduce the accuracy of phylogenetic analyses.

**Table 1:** Common *cox1* primers used in arachnid systematics, and tested in this study.

Name	Primer Sequence	Reference
LCO1490	GGTCAACAAATCATAAAGATATTGG	[33]
HCO2198	TAAACTTCAGGGTGACCAAAAAAT	[33]
C1-J-2183	CAACATTTATTTTGATTTTTT	[30]
CO1-J-1718	GGAGGATTTGGAAATTGATTAGTTCC	[30]
C1-N-2776	GGATAATCAGAATATCGTCGAGG	[28]
dgLCO1490	GGTCAACAAATCATAAAGAYATYGG	[40]
dgHCO2198	TAAACTTCAGGGTGACCAAARAAYCA	[40]
CO1-N-2735	AAAATGTTGAGGGAAAAAATGTTA	[41]
Chelicerate_R2	GGATGGCCAAAAAATCAAAATAAATG	[42]
CO1-RCF1	GTYTCTTCWATAGTWGAAATRGG	[43]
CO1-RCR1	ACAGAAAAYATATGATGRGCYCAYAC	[43]
C1-J-2123	GATCGAAATTTTAATACTTCTTTTTTTGA	This study

The objective of our study was to explore an optimal strategy for extracting and analyzing arachnid DNA focusing on the barcoding and adjacent *cox1* regions. Our work focused on the species richest arachnid lineage, spiders.

Our first goal was to improve an automated DNA extraction protocol. Compared with manual extraction procedures using kits, robotic DNA extraction methods often yield lower quantity of extracted DNA [34]. To maximize its efficiency, we experimentally adjusted an internal robotic DNA extraction program and improved it for acquisition of high concentration of genomic DNA from different quality tissues. Our second goal was to test the performance of commonly used *cox1* primer combinations and to identify the optimal primer set over the major phylogenetic lineages of spiders. We screened and tested the high throughput utility with a single PCR program of ten *cox1* primer pairs. Our third goal was to develop a new *cox1* primer that would produce an indel-free alignment resulting in more accurate phylogenetic analyses.

## Materials and Methods

### Specimens and taxonomic coverage

**Figure 2:**
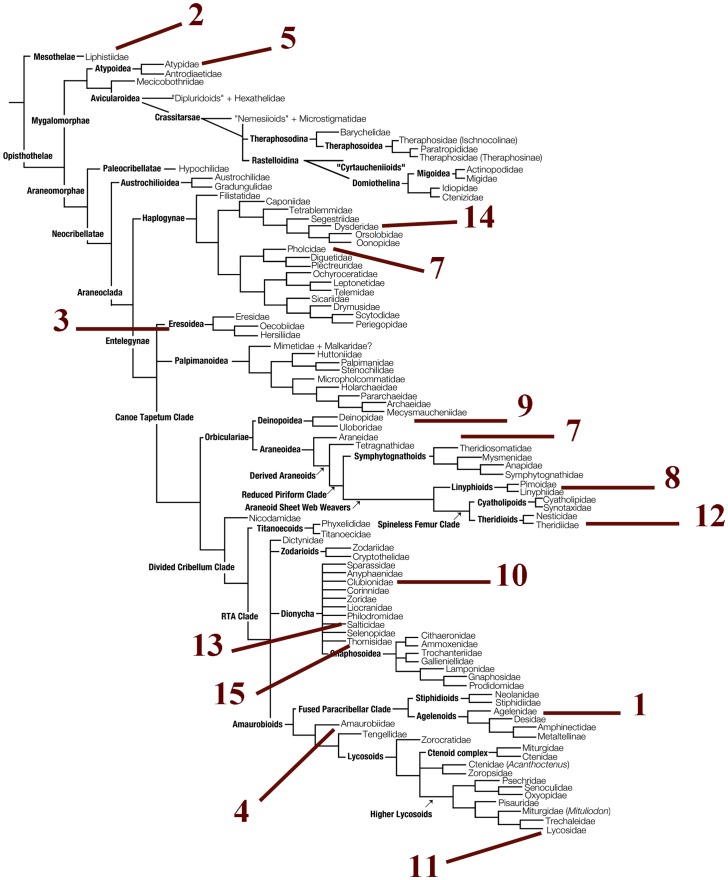
Fifteen selected spider species (see **Table 2**) representing the major phylogenetic lineages on a simplified phylogeny [35].

Fifteen spider species were selected to represent all major spider clades [14], [35] (Table 2; Fig. 2). Specimens were obtained from the EZ Lab tissue bank (http://ezlab.zrc-sazu.si/) for every numbered species and the size of tissue samples varied from 0.3 to 3.0 mm^3^ volume of spider's leg.

**Table 2:** The success rate of different primer combinations for the fifteen selected spider species varies from 100% to 30%.

		Primer pair	LCO-1490	LCO-1490	dgLCO-1490	dgLCO-1490	C1-J-2123	C1-J-2183	CO1-J-1718	CO1-RCF1	CO1-RCF1	LCO-1490
			HCO-2198	Chelicerate_R2	dgHCO-2198	Chelicerate_R2	C1-N-2776	C1-N-2776	CO1-N-2735	CO1-RCR1	CO1-N-2735	C1-N-2776
Sample Nr.	Voucher Nr.	Selected Species (see Tree)										
1	ARA0239	*Agelena labyrinthica*	100%	100%	100%	100%	100%	100%	100%	100%	100%	100%
2	ARA0240	*Liphistius sp.*	100%	100%	100%	100%	/	100%	/	/	/	/
3	ARA0111	*Uroctea durandi*	100%	100%	100%	100%	100%	100%	100%	/	/	50%**
4	ARA0120	*Amaurobius erberi*	100%	100%	100%	100%	100%	100%	100%	100%	100%	100%
5	ARA0174	*Atypus piceus*	100%	100%	100%	100%	100%	100%	/	/	/	/
6	ARA0001	*Araneus angulatus*	100%*	100%	/	100%	100%	100%	100%	100%	100%	/
7	ARA0003	*Pholcus phalangioides*	100%	100%	100%	/	100%	/	/	/	/	/
8	ARA0004	*Linyphia triangularis*	100%	100%	100%	100%	100%	100%	/	/	/	/
9	ARA0241	*Hyptiotes paradoxus*	100%	100%	100%	100%	100%	100%	100%	/	/	/
10	ARA0242	*Clubiona terrestris*	100%	100%	100%	100%	100%	100%	100%	100%	/	/
11	ARA0243	*Pardosa riparia*	100%	100%	100%	100%	100%	100%	100%	100%	100%	/
12	ARA0029	*Steatoda bipunctata*	100%	100%	100%	100%	100%	100%	100%	100%	100%	/
13	ARA0062	*Evarcha arcuata*	100%	100%	100%	100%	100%	100%	100%	100%	100%	100%
14	ARA0244	*Dysdera ninnii*	100%	100%	100%	100%	100%	100%	/	100%	/	/
15	ARA0081	*Misumena vatia*	100%	100%	100%	100%	100%	100%*	100%	100%	100%	100%
**Successful in Nr.**			15	15	14	14	14	14	10	9	7	5
**Success Rate**			**100%**	**100%**	**93%**	**93%**	**93%**	**93%**	**67%**	**60%**	**47%**	**30%**

Voucher numbers refer to EZ Lab (http://ezlab.zrc-sazu.si/) cryo-collection.

*excised gel band used for 2nd PCR; **only C1-N-2776 binds, one way sequence obtained.

### Automated DNA extraction

Robotic DNA extraction was done with MagMAX Express Magnetic Particle Processor (Life Technologies). DNA from muscle cells was extracted from fresh tissue or tissue frozen at -80°C after collection and species identification. DNA was extracted using MagMAX DNA Multi-Sample Kit (Life Technologies) by modifying the manufacturer protocol for manual extraction.

The MagMAX plate was loaded as follows; row A: 80 µL of Multisample DNA Lysis Buffer, 96 µL of Isopropanol, 80 µL of tissue sample in phosphate buffered saline (PBS: 137 mM NaCl, 2.7 mM KCl, 8 mM Na_2_HPO_4_, and 2 mM KH_2_PO_4_, pH 7.4); into row B: 120 µL of Wash Solution I, into row C, E, F: 120 µL of Wash Solution II, into row D: 38 µL of nuclease-free water, into row G: 40 µL of Elution Buffer I. During the run in the MagMAX Express Magnetic Particle Processor the magnetic beads solution (6.4 µL DNA binding beads with 9.6 µL nuclease-free water) into row A and 2 µL of RNase A into row D were added. In second pause 40 µL of Multisample DNA Lysis Buffer and 48 µL of Isopropanol were added into row D. During the third pause a step of incubation in thermoblock at 70°C for 5 min was made. After the incubation, 40 µL of Elution Buffer II was added into row G (from 70 µL down to 30 µL minimum is allowed) and the run continued in the instrument. Samples of purified DNA were transferred to cryovials for storage from row G (see Appendix S1).

The additional step of overnight incubation of starting material with Proteinase K was added to the protocol improving extraction efficiency. Differently sized tissue was cut and thoroughly homogenized with a pestle in a tube with 73.6 µL PK Buffer and 6.4 µL Proteinase K (100 mg/mL), shortly centrifuged and incubated over night at 55°C on a shaker. All reagents and buffers (with exception of PBS) used for DNA extraction are components of MagMAX DNA Multi-Sample Kit (Life Technologies).

During the optimization step with MagMAX DNA Multi-Sample Kit also the comparison with MagMAX Total Nucleic Acid Isolation Kit (Life Technologies) was done (data not shown). After the quantification of extracted nucleic acids with NanoDrop Lite (Thermo Scientific) the amount of extracted DNA was up to 5-fold higher in comparison to sample concentration prepared with MagMAX DNA Multi-Sample Kit. However, better extraction of nucleic acids could also be related to the MagMAX Total Nucleic Acid Isolation Kit specifications where the isolation of all the nucleic acids (ssDNA, dsDNA and RNA) is performed at once. The comparison of material costs showed substantial differences between the kits used in this study, with the MagMAX DNA Multi-Sample Kit being the most economic.

### PCR optimization strategy

For PCR amplification reaction we achieved the best results using GoTaq Flexi Polymerase Kit (Promega) and the cycling program for *cox1* gene. All of the PCR reaction mixtures had a total volume of 25 µL and 1 µL of DNA template (in the range between 3.0 to 50 ng) per reaction was generally used. Each reaction included 5.1 µL of Promega's GoTaq Flexi Buffer, 0.14 µL of GoTaq Flexi Polymerase, 2.5 µL dNTP's (2 mM each), 2.3 µL MgCl_2_ (25 nM), 0.5 µL of each primer (forward and reverse, 20 µM), 0.14 µL BSA (10 mg/mL) and 12.8 µL of sterile distilled water. The thermocycle program for *cox1* amplification consisted of 95°C for 1 min, 5 cycles of 94°C for 40 sec, 45°C for 40 sec and 72°C for 1 min. Additional 35 cycles of 94°C for 40 sec, 51°C for 40 sec and 72°C for 1 min with a final extension at 72°C for 5 min were used. Two samples were amplified with a second round of PCR using the excised band as a template. All reagents and buffers used for PCR were supplied from Promega.

### PCR amplification verification and sequencing

PCR products were stained with Sybr Safe (Invitrogen), separated by standard 1.5% agarose gel using Owl B2 EasyCast Mini Gel Electrophoresis System and visualized by UV gel imager E-box VX2/20LM (Vilber Lourmat). All of the PCRs were repeated three times to verify strong signals obtained in every reaction where specific PCR fragment was generated. Primers (Table 1) were used in PCRs in 10 different combinations: LCO-1490/HCO-2198, LCO-1490/Chelicerate_R2, dgLCO-1490/dgHCO-2198, dgLCO-1490/Chelicerate_R2, C1-J-2123/C1-N-2776, C1-J-2183/C1-N-2776, CO1-J-1718/CO1-N-2735, LCO-1490/C1-N-2776, CO1-RCF1/CO1-N-2735, CO1-RCF1/CO1-RCR1. Samples were sequenced bidirectionally using Standard-Seq method (Macrogen). Sequence data were edited and assembled using Geneious Pro version 5.4 [36] and further handled in Mesquite version 2.74 [37].

### Primer design

In order to construct a new primer that would bind upstream of the C1-J-2183 primer binding site, we searched for the most conserved region in that area. We used the NCBI nucleotide search facility within Geneious Pro to gather all *cox1* sequences of the order *Araneae* that contained the keyword “BARCODE”. This search tagged sequences meeting all CBOL criteria [38]. All sequences longer or shorter than 658 bp were deleted and the remaining 1672 sequences, already aligned, were used for primer design (see Appendix S2). Potential primers were evaluated using the program Primer3 [39].

## Results

We optimized and improved the manufacturer's protocol for extraction of genomic DNA using the MagMAX Express Magnetic Particle Processor, an automated high throughput DNA extraction system. We processed a wide range of spider tissue of different taxonomic affiliation, size and quality, to improve the protocol and increase the efficiency of the procedure. The manufacturer's protocol only specifies the use of the kit with MagMAXExpress-96 Standard Magnetic Particle Processor or manually. Our procedure describes the use of the MagMAX Express Magnetic Particle Processor in combination with the MagMAX DNA Multi-Sample Kit. Additionally, we optimized the workflow for smaller quantities of starting material and accordingly adjusted and modified the internal program. Changes were made to the volume of reagents used and timing of specific steps (see Appendix S1).

**Figure 3:**
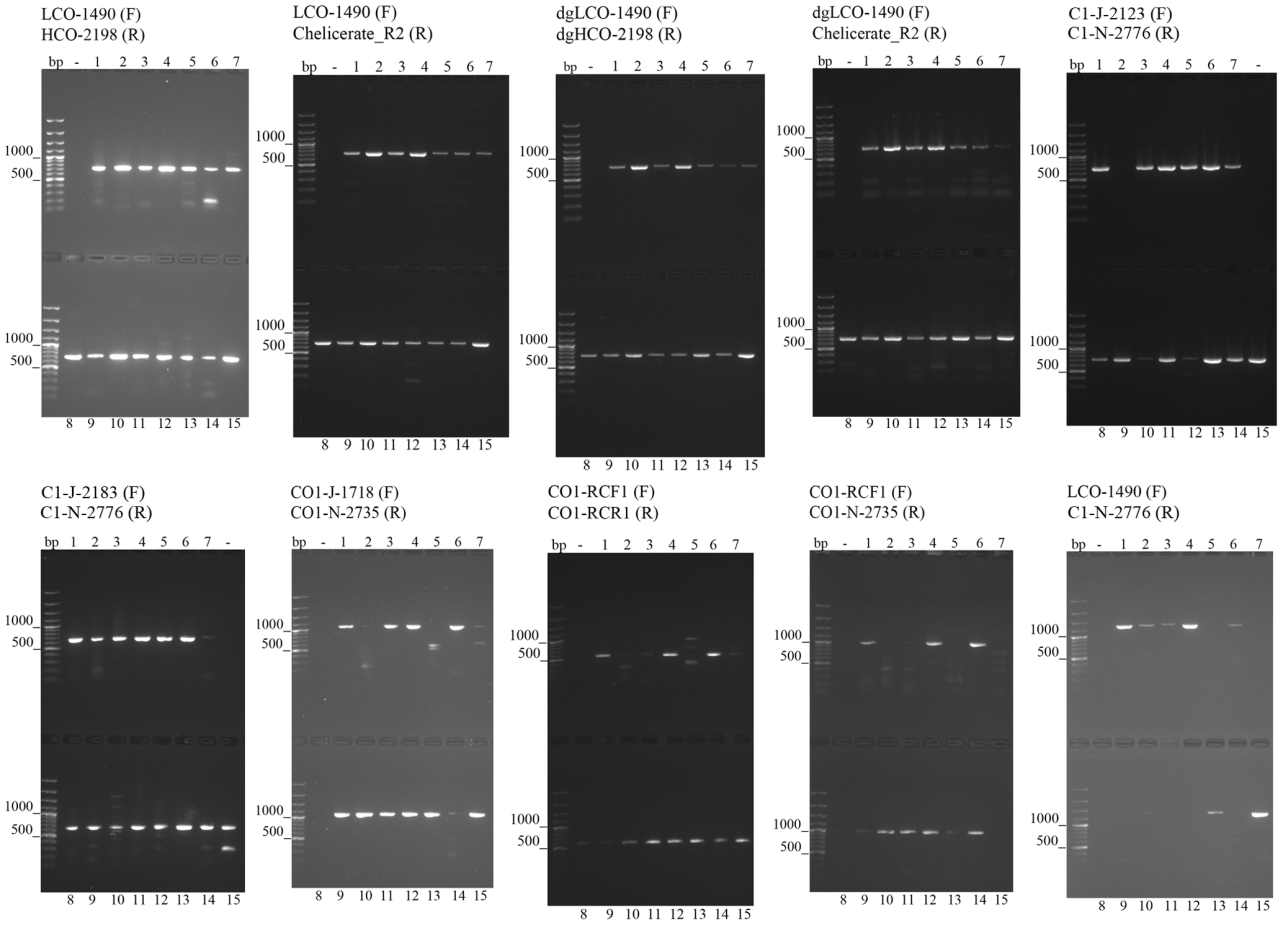
Gel images showing different success rates in *cox1* amplification using the ten tested primer pairs.

To assess the amplification success of ten primer pairs routinely employed for barcoding *cox1* gene, we tested fifteen target species throughout the spider phylogeny [14], [35] (Fig. 2; Table 2) and performed PCR reactions using ten primer pairs (Table 2). Our goal was to compare the performance of each primer pair against every representative across the tree and evaluate their success rate. The success rate of different primer pairs varied from 30 to 100% (Fig. 3; Table 2). To target solely the short barcoding *cox1* region, we recommend using the combination of Folmer primers (LCO1490/HCO2198) [33].

**Figure 4:**
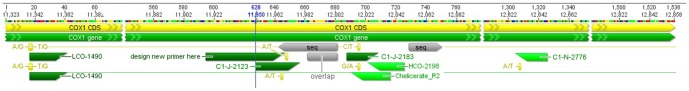
The new primer C1-J-2123 binding site is 60 bp upstream of the C1-J-2183 binding site.

To maximize sequence data for genetic, genomic, and phylogenetic analyses, however, a longer stretch of *cox1* is desired. Existing primers can fail to provide a continuous stretch if used in a single pair or in combinations of pairs [12], [31]–[32]. The forward primer C1-J-2183, for example, is designed too close to the reverse HCO2198 for accurate chromatogram reads, and in practice the resulting interpretations of base pairs result in two partial *cox1* sequences (Fig. 1). We therefore designed, via analysis of the consensus alignment of 1672 arachnid *cox1* sequences a new forward DNA primer situated 60 base pairs upstream of the C1-J-2183 binding site. The sequence GATCGAAATTTTAATACTTCTTTTTTTGA was chosen as the most conserved and appropriate for the binding of a new primer, named C1-J-2123 (Fig. 4). Our preliminary tests (data not shown) suggest that this primer will work not only in spiders, but also other arachnids (scorpions, mites and ticks) and other invertebrates (bivalves, gastropods, tunicates and others).

**Figure 5:**
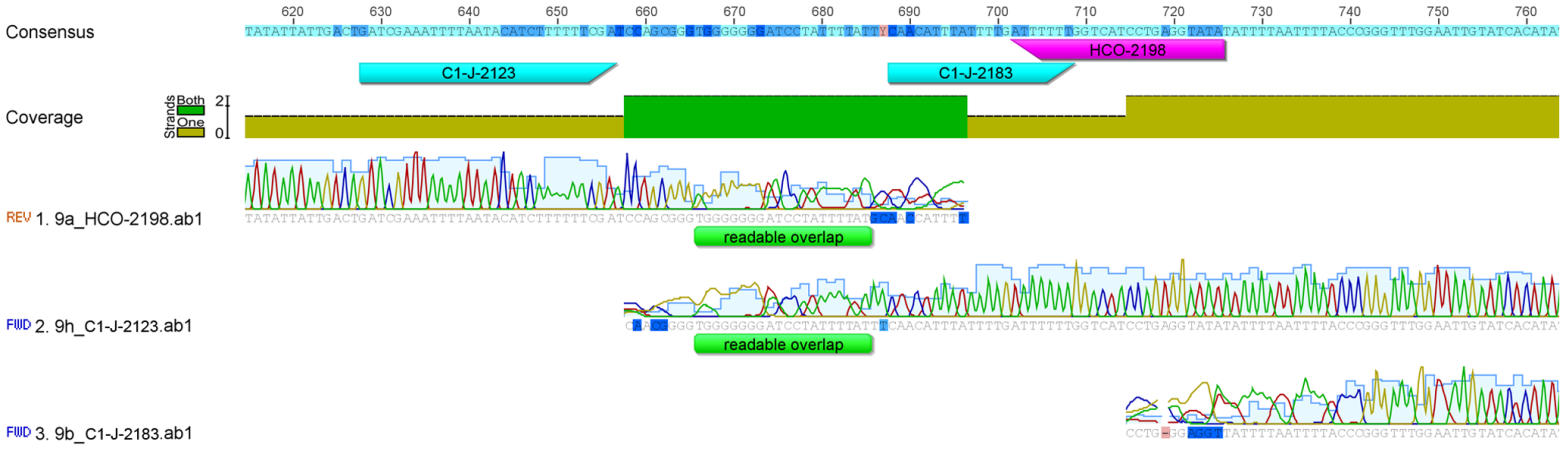
The newly amplified and elongated C1-J-2123/C1-N-2776 sequence overlaps with the Folmer (LCO1490/HCO2198) sequence.

C1-J-2123 performed with the same success rate (93%) as the alternative primer C1-J-2183, amplifying in 14 out of 15 spider species (Fig. 3; Table 2). We recommend using the C1-J-2123/C1-N-2776 primer pair extended in upstream direction, which will allow for full overlap of this extended sequence with that obtained with the standard Folmer primers LCO1490 and HCO2198. *Cox1* sequences obtained with the C1-J-2123/C1-N-2776 primer pair can fully sequence both regions (Fig. 5).

## Conclusions

This study assessed the usefulness, measured as amplification success, of ten primer pairs routinely employed for targeting the barcoding and other *cox1* gene regions in arachnids. Aiming to optimize the efforts in pursuing a longer stretch of *cox1* that would maximize the data versus effort ratio for phylogenetic use of the barcode data, we sought an ideal protocol for automatic, reliable and fast extraction of genomic DNA, and developed a new *cox1* primer for routine spider systematic work. Our newly designed *cox1* primer C1-J-2123 replaces C1-J-2183 to avoid creating an indel region after the Folmer region. This may be especially useful to obtain more complete *cox1* data for phylogenetic analyses.

We also improved the robotic DNA extraction protocol from the manufacturer's version, adapting it for spider tissue. We are the first to convey usage and set protocol for DNA extraction with MagMAX Express Magnetic Particle Processor in combination with MagMAX DNA Multi-Sample Kit. Our protocol allows for higher DNA concentration output compared with manual DNA extraction using commercial kits. It is thus suitable for semi-high throughput preparation of arachnid DNA. With the 1.5 hour DNA extraction run, the system can be loaded about 8 times per day providing DNA isolated from 192 samples. Following our protocol, PCR amplification of 96 samples is possible in only two hours using a single PCR program. Our protocol is fast and effective and able to provide up to 1000 amplifications per week. Using an even higher throughput system such as the MagMAX Express-96 Magnetic Particle Processor that processes 96 samples at a time, this time could be further cut in half.

## Supporting Information

Appendix S1 **Internal Program of MagMAX Express DNA Extraction Robot (Life Technologies) Protocol, modified.** See separate file.
(DOCX)

Appendix S2 **Final DNA sequence assembly accession information.** See separate file.
(DOCX)
