# Supplementary material for: Streamlining DNA Barcoding Protocols: Automated DNA Extraction and a New cox1 Primer in Arachnid Systematics
Source: PLoS One. 2014 Nov 21;9(11):e113030. doi: 10.1371/journal.pone.0113030 (PMC4240537; doi:10.1371/journal.pone.0113030)
Supplement: Appendix S1 — Internal Program of MagMAX Express DNA Extraction Robot (Life Technologies) Protocol, modified. See separate file. (DOCX) [file pone.0113030.s001.docx]

Appendix S1.

Separate steps of DNA extraction using MultiSample DNA kit (Life Technologies) were adjusted to increase the concentration of extracted DNA.

PROTOCOL STEPS

1. Mixing step. Sample of homogenized tissue preincubated with Proteinase K is mixed for 4 min in well A. During the pause, 16 uL of bead mix is added into each well of row A. 2 µL of RNase A is added into row D.

2. Mixing step. The solution is mixed for 4 min in wells of row A with slow speed. Beads are collected at the end of step.

3. Binding step. The solution is incubated for 5 min and mixed with very slow speed to allow binding of DNA to the beads. Beads are collected at the end of step and transferred to wells of row B.

4. Washing step. Beads are released in 10 s with fast speed to wells of row B and washed for 30 s with medium speed.

5. Binding step. The solution is incubated for 5 min and mixed with very slow speed to allow binding of DNA to the beads. Beads are collected at the end of step and transferred to wells of row C.

6. Washing step. Beads are released in 10 s with fast speed to wells of row C and washed again for 30 s with medium speed. Beads are collected at the end of step.

7. Binding step. The solution is incubated for 3 min and mixed with very slow speed to allow binding of DNA to the beads. Beads are collected at the end of step.

8. Drying step. The beads collected at the magnetic tip are left outside the well to dry for 3 min.

9. Mixing step. The solution of RNase A is mixed for 3 min in wells of row D. Beads are released in 10 s with fast speed. The solution is mixed for 3 min with fast speed. During the pause, Multisample DNA Lysis Buffer and Isopropanol are added into row D.

10. Mixing step. The solution is mixed for 4 min in wells of row D with fast speed. Beads are collected at the end of step.

11. Binding step. Beads are released in 10 s with fast speed to wells of row D. The solution is incubated for 3 min and mixed with very slow speed to allow binding of DNA to the beads. Beads are collected at the end of step and transferred to wells of row E.

12. Washing step. Beads are released in 10 s with fast speed to wells of row E and washed for 30 s with medium speed. Beads are collected at the end of step.

13. Binding step. The solution is incubated for 3 min and mixed with very slow speed to allow binding of DNA to the beads. Beads are collected at the end of step and transferred to wells of row F.

14. Washing step. Beads are released in 10 s with fast speed to wells of row F and washed for 30 s with medium speed. Beads are collected at the end of step.

15. Binding step. The solution is incubated for 3 min and mixed with very slow speed to allow binding of DNA to the beads. Beads are collected at the end of step.

16. Drying step. The beads collected at the magnetic tip are left outside the well to dry for 3 min.

17. Mixing step. Beads are released in 3 min with very slow speed to wells of row G and the solution is mixed for 2 min in wells of row G with slow speed. During the pause, the plate is incubated for 5 min at 70 °C. Elution Buffer II is added into wells of row G.

18. Mixing step. Beads are released in 20 s with fast speed to wells of row G and the solution is mixed for 30 s in wells of row G with slow speed.

19. Elution step. Beads are released in 30 s with slow speed to wells of row G and the solution is eluted from beads for 5 min with slow speed. Beads are removed and disposed to wells of row B.

20. Solution of purified DNA is collected from row G.
