# Supplementary material for: Streamlining DNA Barcoding Protocols: Automated DNA Extraction and a New cox1 Primer in Arachnid Systematics
Source: PLoS One. 2014 Nov 21;9(11):e113030. doi: 10.1371/journal.pone.0113030 (PMC4240537; doi:10.1371/journal.pone.0113030)
Supplement: Appendix S2 — Final DNA sequence assembly accession information. See separate file. (DOCX) [file pone.0113030.s002.docx]

Appendix S2.

GU682436, GU682439, GU682440, GU682441, GU682442, GU682443, GU682444, GU682445, GU682446, GU682447, GU682448, GU682449, GU682450, GU682451, GU682452, GU682453, GU682456, GU682457, GU682458, GU682459, GU682460, GU682461, GU682462, GU682463, GU682465, GU682466, GU682467, GU682468, GU682469, GU682470, GU682471, GU682472, GU682474, GU682475, GU682476, GU682477, GU682478, GU682479, GU682480, GU682481, GU682482, GU682483, GU682484, GU682485, GU682486, GU682487, GU682488, GU682490, GU682491, GU682492, GU682494, GU682496, GU682497, GU682498, GU682499, GU682500, GU682501, GU682502, GU682503, GU682504, GU682505, GU682506, GU682507, GU682508, GU682510, GU682511, GU682512, GU682513, GU682514, GU682515, GU682517, GU682518, GU682519, GU682520, GU682522, GU682523, GU682524, GU682525, GU682526, GU682527, GU682528, GU682529, GU682530, GU682531, GU682532, GU682533, GU682534, GU682535, GU682536, GU682537, GU682538, GU682539, GU682541, GU682542, GU682543, GU682544, GU682545, GU682549, GU682550, GU682551, GU682552, GU682553, GU682554, GU682555, GU682556, GU682557, GU682559, GU682560, GU682561, GU682562, GU682563, GU682564, GU682565, GU682566, GU682567, GU682568, GU682570, GU682571, GU682572, GU682573, GU682574, GU682575, GU682576, GU682577, GU682578, GU682579, GU682580, GU682581, GU682582, GU682583, GU682585, GU682586, GU682587, GU682588, GU682589, GU682590, GU682591, GU682592, GU682593, GU682595, GU682596, GU682597, GU682598, GU682600, GU682601, GU682602, GU682603, GU682604, GU682605, GU682606, GU682607, GU682608, GU682609, GU682611, GU682612, GU682613, GU682614, GU682615, GU682616, GU682617, GU682618, GU682619, GU682620, GU682621, GU682622, GU682623, GU682624, GU682625, GU682626, GU682627, GU682628, GU682629, GU682630, GU682632, GU682634, GU682635, GU682637, GU682638, GU682639, GU682640, GU682641, GU682642, GU682643, GU682644, GU682645, GU682646, GU682647, GU682648, GU682649, GU682651, GU682652, GU682653, GU682654, GU682656, GU682658, GU682659, GU682660, GU682661, GU682662, GU682663, GU682664, GU682665, GU682666, GU682667, GU682668, GU682669, GU682670, GU682671, GU682672, GU682674, GU682675, GU682676, GU682677, GU682678, GU682679, GU682680, GU682681, GU682682, GU682683, GU682684, GU682685, GU682686, GU682687, GU682688, GU682689, GU682690, GU682691, GU682692, GU682693, GU682694, GU682695, GU682696, GU682697, GU682698, GU682699, GU682700, GU682701, GU682702, GU682703, GU682704, GU682705, GU682706, GU682707, GU682708, GU682709, GU682710, GU682711, GU682712, GU682713, GU682714, GU682715, GU682717, GU682718, GU682719, GU682720, GU682721, GU682722, GU682723, GU682724, GU682725, GU682726, GU682728, GU682732, GU682733, GU682734, GU682735, GU682736, GU682737, GU682738, GU682739, GU682740, GU682741, GU682742, GU682743, GU682744, GU682745, GU682746, GU682747, GU682748, GU682749, GU682750, GU682751, GU682752, GU682753, GU682754, GU682755, GU682756, GU682757, GU682758, GU682759, GU682760, GU682761, GU682762, GU682763, GU682764, GU682765, GU682766, GU682767, GU682768, GU682769, GU682770, GU682771, GU682772, GU682773, GU682774, GU682775, GU682776, GU682777, GU682778, GU682779, GU682780, GU682781, GU682782, GU682784, GU682785, GU682786, GU682787, GU682788, GU682789, GU682790, GU682791, GU682792, GU682793, GU682794, GU682795, GU682796, GU682797, GU682798, GU682799, GU682800, GU682801, GU682802, GU682803, GU682804, GU682805, GU682806, GU682807, GU682808, GU682809, GU682811, GU682812, GU682813, GU682814, GU682815, GU682816, GU682817, GU682819, GU682820, GU682821, GU682822, GU682823, GU682824, GU682825, GU682826, GU682827, GU682828, GU682830, GU682831, GU682832, GU682833, GU682834, GU682835, GU682836, GU682837, GU682838, GU682839, GU682840, GU682841, GU682842, GU682843, GU682844, GU682845, GU682846, GU682847, GU682848, GU682849, GU682850, GU682851, GU682852, GU682853, GU682854, GU682855, GU682856, GU682857, GU682858, GU682859, GU682860, GU682861, GU682862, GU682863, GU682864, GU682865, GU682866, GU682867, GU682868, GU682869, GU682870, GU682871, GU682872, GU682873, GU682874, GU682875, GU682876, GU682877, GU682878, GU682879, GU682880, GU682881, GU682882, GU682883, GU682884, GU682885, GU682886, GU682888, GU682889, GU682893, GU682895, GU682896, GU682897, GU682898, GU682899, GU682900, GU682901, GU682902, GU682903, GU682904, GU682905, GU682906, GU682907, GU682908, GU682909, GU682912, GU682913, GU682914, GU682915, GU682916, GU682917, GU682918, GU682919, GU682921, GU682922, GU682923, GU682924, GU682925, GU682926, GU682927, GU682928, GU682929, GU682930, GU682931, GU682932, GU682933, GU682934, GU682935, GU682937, GU682938, GU682939, GU682941, GU682942, GU682945, GU682946, GU682947, GU682948, GU682949, GU682950, GU682951, GU682952, GU682953, GU682954, GU682955, GU682956, GU683113, GU683594, GU683595, GU683597, GU683598, GU683599, GU683600, GU683601, GU683602, GU683603, GU683604, GU683606, GU683607, GU683608, GU683609, GU683610, GU683611, GU683612, GU683613, GU683614, GU683616, GU683621, GU683622, GU683623, GU683628, GU683629, GU683630, GU683638, GU683642, GU683643, GU683644, GU683649, GU683650, GU683651, GU683652, GU683653, GU683656, GU683660, GU683661, GU683663, GU683668, GU683669, GU683671, GU683672, GU683673, GU683674, GU683675, GU683677, GU683678, GU683681, GU683689, GU683691, GU683692, GU683694, GU683695, GU683696, GU683697, GU683699, GU683700, GU683701, GU683703, GU683706, GU683707, GU683708, GU683709, GU683711, GU683713, GU683714, GU683715, GU683716, GU683717, GU683718, GU683719, GU683720, GU683721, GU683724, GU683725, GU683726, GU683727, GU683728, GU683730, GU683732, GU683735, GU683736, GU683737, GU683738, GU683740, GU683741, GU683743, GU683745, GU683746, GU683747, GU683748, GU683750, GU683751, GU683752, GU683754, GU683755, GU683767, GU683769, GU683770, GU683771, GU683772, GU683773, GU683774, GU683775, GU683776, GU683777, GU683778, GU683779, GU683780, GU683781, GU683782, GU683785, GU683786, GU683787, GU683788, GU683789, GU683790, GU683791, GU683792, GU683793, GU683794, GU683796, GU683798, GU683799, GU683800, GU683801, GU683802, GU683803, GU683805, GU683806, GU683807, GU683808, GU683811, GU683812, GU683813, GU683814, GU683815, GU683817, GU683818, GU683819, GU683820, GU683821, GU683822, GU683823, GU683824, GU683825, GU683827, GU683834, GU683835, GU683837, GU683840, GU683842, GU683843, GU683844, GU683845, GU683846, GU683848, GU683849, GU683850, GU683851, GU683852, GU683853, GU683854, GU683855, GU683856, GU683857, GU683858, GU683859, GU683860, GU683861, GU683862, GU683863, GU683864, GU683865, GU683866, GU683867, GU683868, GU683871, GU683872, GU683873, GU683874, GU683878, GU683879, GU683880, GU683882, GU683883, GU683885, GU683887, GU683888, GU683889, GU683891, GU683893, GU683894, GU683898, GU683899, GU683900, GU683901, GU683902, GU683903, GU683904, GU683905, GU683906, GU683907, GU683908, GU683909, GU683910, GU683911, GU683916, GU683917, GU683919, GU683920, GU683921, GU683922, GU683923, GU683924, GU683925, GU683926, GU683927, GU683928, GU683929, GU683930, GU683931, GU683932, GU683933, GU683934, GU683935, GU683936, GU683937, GU683938, GU683939, GU683940, GU683941, GU683942, GU683943, GU683944, GU683945, GU683946, GU683947, GU683948, GU683949, GU683950, GU683951, GU683952, GU683953, GU683954, GU683955, GU683956, GU683957, GU683958, GU683959, GU683960, GU683961, GU683962, GU683963, GU683964, GU683965, GU683966, GU683967, GU683968, GU683969, GU683970, GU683971, GU683972, GU683973, GU683974, GU683975, GU683976, GU683977, GU683978, GU683979, GU683980, GU683981, GU683982, GU683983, GU683984, GU683985, GU683986, GU683987, GU683988, GU683989, GU683990, GU683992, GU683993, GU683994, GU683999, GU684001, GU684002, GU684003, GU684004, GU684005, GU684006, GU684007, GU684008, GU684009, GU684010, GU684011, GU684012, GU684013, GU684014, GU684015, GU684016, GU684017, GU684025, GU684026, GU684027, GU684031, GU684032, GU684045, GU684082, GU684101, GU684104, GU684106, GU684110, GU684112, GU684114, GU684118, GU684119, GU684120, GU684123, GU684149, GU684150, GU684151, GU684160, GU684170, GU684185, GU684186, GU684187, GU684188, GU684189, GU684190, GU684191, GU684192, GU684193, GU684194, GU684195, GU684196, GU684197, GU684198, GU684199, GU684201, GU684202, GU684203, GU684204, GU684205, GU684206, GU684207, GU684208, GU684209, GU684210, GU684211, GU684212, GU684213, GU684214, GU684215, GU684216, GU684217, GU684218, GU684219, GU684220, GU684221, GU684222, GU684223, GU684224, GU684225, GU684226, GU684227, GU684228, GU684229, GU684230, GU684231, GU684232, GU684233, GU684234, GU684235, GU684236, GU684237, GU684238, GU684239, GU684240, GU684241, GU684242, GU684243, GU684244, GU684245, GU684246, GU684247, GU684248, GU684249, GU684250, GU684251, GU684252, GU684253, GU684254, GU684255, GU684256, GU684257, GU684258, GU684259, GU684260, GU684261, GU684262, GU684265, GU684266, GU684267, GU684268, GU684269, GU684270, GU684271, GU684272, GU684273, GU684297, GU684298, GU684300, GU684301, GU684302, GU684304, GU684307, GU684309, GU684316, GU684317, GU684319, GU684320, GU684321, GU684328, GU684331, GU684335, GU684340, GU684341, GU684343, GU684345, GU684347, GU684354, GU684355, GU684356, GU684357, GU684358, GU684360, GU684361, GU684363, GU684364, GU684365, GU684366, GU684367, GU684368, GU684369, GU684372, GU684373, GU684375, GU684379, GU684380, GU684381, GU684383, GU684387, GU684388, GU684389, GU684391, GU684392, GU684393, GU684394, GU684395, GU684396, GU684397, GU684398, GU684399, GU684400, GU684401, GU684402, GU684403, GU684404, GU684405, GU684406, GU684407, GU684408, GU684412, GU684414, GU684416, GU684417, GU684418, GU684419, GU684423, GU684425, GU684426, GU684428, GU684429, GU684430, GU684431, GU684432, GU684435, GU684436, GU684437, GU684438, GU684440, GU684441, GU684442, GU684443, GU684445, GU684454, GU684455, GU684457, GU684458, GU684459, GU684461, GU684463, GU684464, GU684465, GU684466, GU684468, GU684469, GU684472, GU684473, GU684474, GU684475, GU684476, GU684477, GU684478, GU684479, GU684480, GU684481, GU684483, GU684484, GU684485, GU684486, GU684487, GU684489, GU684490, GU684491, GU684492, GU684493, GU684494, GU684496, GU684497, GU684498, GU684499, GU684500, GU684501, GU684503, GU684504, GU684505, GU684506, GU684508, GU684509, GU684510, GU684511, GU684516, GU684517, GU684518, GU684519, GU684521, GU684522, GU684524, GU684525, GU684526, GU684528, GU684529, GU684530, GU684532, GU684535, GU684536, GU684537, GU684545, GU684547, GU684548, GU684549, GU684550, GU684551, GU684552, GU684553, GU684554, GU684555, GU684558, GU684562, GU684563, GU684564, GU684565, GU684566, GU684567, GU684568, GU684570, GU684573, GU684574, GU684575, GU684576, GU684577, GU684578, GU684579, GU684580, GU684581, GU684583, GU684584, GU684585, GU684586, GU684587, GU684588, GU684589, GU684590, GU684591, GU684592, GU684593, GU684594, GU684595, GU684596, GU684597, GU684598, GU684599, GU684600, GU684601, GU684603, GU684604, GU684605, GU684606, GU684607, GU684608, GU684609, GU684610, GU684611, GU684612, GU684613, GU684614, GU684615, GU684616, GU684617, GU684620, GU684621, GU684622, GU684623, GU684624, GU684625, GU684626, GU684627, GU684628, GU684629, GU684630, GU684631, GU684632, GU684633, GU684634, GU684635, GU684636, GU684637, GU684638, GU684639, GU684640, GU684641, GU684643, GU684644, GU684645, GU684646, GU684647, GU684648, GU684649, GU684650, GU684652, GU684653, GU684654, GU684655, GU684656, GU684657, GU684658, GU684659, GU684660, GU684661, GU684662, GU684663, GU684664, GU684665, GU684666, GU684667, GU684668, GU684670, GU684671, GU684672, GU684673, GU684674, GU684676, GU684679, GU684680, GU684681, GU684682, GU684684, GU684686, GU684687, GU684688, GU684689, GU684691, GU684695, GU684696, GU684698, GU684699, GU684700, GU684701, GU684702, GU684704, GU684706, GU684707, GU684708, GU684709, GU684710, GU684711, GU684712, GU684713, GU684714, GU684716, GU684717, GU684718, GU684720, GU684721, GU684722, GU684723, GU684724, GU684725, GU684727, GU684729, GU684730, GU684731, GU684732, GU684733, GU684734, GU684735, GU684736, GU684738, GU684739, GU684741, GU684743, HM376095, HM376096, HM376097, HM376098, HM376099, HM376102, HM376103, HM376104, HM376105, HM376106, HM376107, HM377208, HM416913, HM416916, HM432622, HM432623, HM432624, HM432626, HM432627, HM432628, HM432631, HM432633, HM432634, HM434049, HM434050, HM434051, HM434052, HM434053, HM434054, HM434056, HM434057, HM434059, HM434060, HM434061, HM434062, HM434063, HM434064, HM434065, HM434066, HM434067, HM434070, HM880614, HM880619, HM880620, HM880621, HM880622, HM880623, HM880625, HM880626, HM880627, HQ924378, HQ924379, HQ924380, HQ924381, HQ924382, HQ924383, HQ924384, HQ924385, HQ924386, HQ924387, HQ924388, HQ924389, HQ924390, HQ924391, HQ924392, HQ924393, HQ924394, HQ924395, HQ924396, HQ924397, HQ924398, HQ924399, HQ924400, HQ924401, HQ924402, HQ924403, HQ924404, HQ924405, HQ924406, HQ924407, HQ924408, HQ924409, HQ924410, HQ924411, HQ924412, HQ924413, HQ924414, HQ924415, HQ924416, HQ924417, HQ924418, HQ924419, HQ924420, HQ924421, HQ924422, HQ924423, HQ924424, HQ924425, HQ924426, HQ924427, HQ924428, HQ924429, HQ924430, HQ924431, HQ924432, HQ924433, HQ924434, HQ924435, HQ924436, HQ924437, HQ924438, HQ924439, HQ924440, HQ924441, HQ924442, HQ924443, HQ924444, HQ924445, HQ924446, HQ924447, HQ924448, HQ924449, HQ924450, HQ924451, HQ924452, HQ924453, HQ924454, HQ924455, HQ924456, HQ924458, HQ924459, HQ924460, HQ924461, HQ924462, HQ924463, HQ924464, HQ924465, HQ924466, HQ924467, HQ924468, HQ924470, HQ924471, HQ924472, HQ924473, HQ924474, HQ924475, HQ924476, HQ924477, HQ924479, HQ924480, HQ924481, HQ924482, HQ924483, HQ924484, HQ924485, HQ924486, HQ924487, HQ924488, HQ924489, HQ924490, HQ924491, HQ924492, HQ924494, HQ924495, HQ924496, HQ924497, HQ924498, HQ924499, HQ924500, HQ924501, HQ924502, HQ924503, HQ924504, HQ924506, HQ924507, HQ924508, HQ924509, HQ924510, HQ924511, HQ924512, HQ924513, HQ924514, HQ924515, HQ924516, HQ924517, HQ924518, HQ924519, HQ924520, HQ924521, HQ924522, HQ924523, HQ924524, HQ924526, HQ924527, HQ924528, HQ924529, HQ924530, HQ924533, HQ924534, HQ924535, HQ924536, HQ924537, HQ924538, HQ924539, HQ924540, HQ924541, HQ924542, HQ924543, HQ924544, HQ924545, HQ924546, HQ924547, HQ924548, HQ924549, HQ924550, HQ924551, HQ924552, HQ924553, HQ924554, HQ924555, HQ924556, HQ924557, HQ924560, HQ924562, HQ924563, HQ924564, HQ924565, HQ924566, HQ924567, HQ924568, HQ924569, HQ924570, HQ924571, HQ924572, HQ924573, HQ924574, HQ924575, HQ924576, HQ924577, HQ924578, HQ924579, HQ924580, HQ924581, HQ924582, HQ924583, HQ924584, HQ924585, HQ924586, HQ924587, HQ924588, HQ924589, HQ924590, HQ924591, HQ924592, HQ924593, HQ924594, HQ924595, HQ924597, HQ924598, HQ924599, HQ924600, HQ924601, HQ924602, HQ924604, HQ924605, HQ924606, HQ924607, HQ924609, HQ924610, HQ924611, HQ924612, HQ924613, HQ924614, HQ924615, HQ924616, HQ924618, HQ924619, HQ924620, HQ924621, HQ924625, HQ924626, HQ924628, HQ924629, HQ924631, HQ924632, HQ924633, HQ924634, HQ924635, HQ924636, HQ924637, HQ924638, HQ924639, HQ924640, HQ924641, HQ924642, HQ924643, HQ924644, HQ924645, HQ924646, HQ924647, HQ924648, HQ924649, HQ924650, HQ924651, HQ924652, HQ924653, HQ924654, HQ924655, HQ924656, HQ924657, HQ924658, HQ924659, HQ924660, HQ924661, HQ924662, HQ924663, HQ924664, HQ924665, HQ924666, HQ924667, HQ924668, HQ928191, HQ979175, HQ979176, HQ979177, HQ979178, HQ979179, HQ979180, HQ979181, HQ979182, HQ979183, HQ979206, HQ979207, HQ979208, HQ979209, HQ979210, HQ979212, HQ979213, HQ979215, HQ979216, HQ979217, HQ979218, HQ979219, HQ979220, HQ979221, HQ979222, HQ979223, HQ979224, HQ979225, HQ979226, HQ979227, HQ979228, HQ979229, HQ979230, HQ979231, HQ979232, HQ979233, HQ979251, HQ979252, HQ979253, HQ979254, HQ979255, HQ979256, HQ979257, HQ979258, HQ979259, HQ979260, HQ979261, HQ979263, HQ979264, HQ979265, HQ979266, HQ979267, HQ979268, HQ979269, HQ979270, HQ979271, HQ979272, HQ979273, HQ979274, HQ979275, HQ979276, HQ979277, HQ979278, HQ979279, HQ979280, HQ979281, HQ979282, HQ979283, HQ979284, HQ979285, HQ979286, HQ979287, HQ979288, HQ979289, HQ979290, HQ979291, HQ979292, HQ979293, HQ979294, HQ979295, HQ979296, HQ979297, HQ979298, HQ979299, HQ979300, HQ979301, HQ979302, HQ979303, HQ979304, HQ979305, HQ979306, HQ979307, HQ979308, HQ979309, HQ979310, HQ979311, HQ979312, HQ979313, HQ979314, HQ979315, HQ979316, HQ979317, HQ979318, HQ979319, HQ979320, HQ979321, HQ979322, HQ979323, HQ979324, HQ979325, HQ979326, HQ979327, HQ979328, HQ979329, HQ979330, HQ979331, HQ979332, HQ979333, HQ979334, HQ979335, HQ979336, HQ979337, HQ979338, HQ979339, HQ979340, HQ979341, HQ979342, HQ979343, HQ979345, HQ979346, HQ979347, HQ979348, HQ979349, HQ979350, HQ979351, HQ979352, HQ979355, HQ979356, HQ979357, HQ979358, HQ979359, HQ979360, HQ979361, HQ979362, HQ979363, HQ979364, HQ979365, HQ979366, HQ979367, HQ979368, HQ979369, HQ979370, HQ979371, HQ979374, HQ979375.
